# Bile metabolites of polycyclic aromatic hydrocarbons (PAHs) in three species of fish from Morocco

**DOI:** 10.1186/s12302-016-0093-6

**Published:** 2016-11-02

**Authors:** Ayoub Baali, Ulrike Kammann, Reinhold Hanel, Ikram El Qoraychy, Ahmed Yahyaoui

**Affiliations:** 1 Laboratory of Zoology and General Biology, Faculty of Science, Mohammed V University in Rabat, Rabat, Morocco; 2 Thünen Institute of Fisheries Ecology, Hamburg, Germany

**Keywords:** PAH metabolites, Fish, Lagoon, Coast, Morocco

## Abstract

**Background:**

Polycyclic aromatic hydrocarbons (PAH) are environmental contaminants that pose significant risk to health of fish. Environmental pollution of fish is a topic of rising attention in Morocco. However, only few studies have been carried out so far, describing the potential threat of organic pollution to Moroccan aquatic ecosystem. Two polycyclic aromatic hydrocarbon (PAH) metabolites, 1-hydroxypyrene (1-OH-Pyr) and 1-hydroxyphenanthrene (1-OH-Phen), were identified and quantified from the bile of 18 European eels (*Anguilla anguilla*), 7 Moray (*Muraenidae*), and 28 Conger eels (*Conger conger*) collected from Moulay Bousselham lagoon and Boujdour coast. The bile metabolites were separated by high-performance liquid chromatography with fluorescence detection. The present study aims to compare the levels of PAH metabolites in fish from the lagoon and the open sea and to compare levels of PAH metabolites in different fish species.

**Results:**

The major metabolite present in all fish was 1-hydroxypyrene (<LOD-15.56 ng/mL) with lower concentration of 1-hydroxyphenanthrene (<LOD-9.6 ng/mL). These concentrations of PAH metabolites are low compared to studies published before.

**Conclusion:**

The data confirm the importance of 1-hydroxypyrene as the key PAH metabolite in fish bile and suggest that the European eel is an ideal species for monitoring PAHs in Moroccan waters. The present study provides valuable information on concentrations of PAH metabolites in fish from Morocco, especially for the first time for Conger eels and Moray.

## Background

The European conger eel (*Conger conger* L. 1758) is distributed in the northeastern Atlantic, the Mediterranean Sea and the western Black Sea [1].

It is a benthic fish found on rocky and sandy bottoms [2] living in over 1000 m depth [3]. The European conger eel is an important commercial and recreational fishing species of the northeastern Atlantic and the Mediterranean Sea. It is caught mainly by catch in bottom trawl and demersal long-line fisheries targeting ground fish and deep-water species and is also caught by rod and line [4].

Despite being a geographically widespread species and a commercial resource, the number of studies on this species is very limited.

Environmental pollution of fish is a topic of rising attention in Morocco [4, 5]. However, only few studies have been carried out so far, describing the potential threat of organic pollution to Moroccan aquatic ecosystem [6–11]. PAH are ubiquitous environmental contaminants found in marine sediments and waters associated with urbanized estuarine and coastal pollution as well as in rivers [12, 13].

PAH are derived from both natural and anthropogenic sources. The latter can be related to pyrolysis and incomplete combustion of organic matter [14]. Natural sources for PAHs are forest fires and degradation of biological materials, which have led to the presence of these compounds in sediments and to the formation of fossil fuels [14]. For the aquatic environment wastewater, atmospheric deposition and petroleum spillage are further prominent sources. PAHs and their intermediate degradation products have the potential to induce toxic or mutagenic effects in fish [15–17] and humans [18].

PAH metabolites in the bile fluid are widely accepted as measures for PAH exposure in fish because of the rapid metabolism of PAH in most vertebrates [13]. As a consequence, PAH metabolites in fish are recommended as monitoring parameters in European Seas [19, 20]. High-performance liquid chromatography (HPLC) is widely used for the determination of PAH metabolites in different fish species [21–25] and has been covered by an intercalibration exercise [26].

Teleost fish have a high capacity to metabolize PAHs because of cytochrome P-450 enzymes in their tissues that oxidatively biotransform PAHs to hydroxylated metabolites. The cytochrome P-450-dependent enzyme system is often referred to as phase I metabolism. Teleost fish also have well-developed phase II enzyme systems that can make the hydroxylated metabolites more water soluble.

Cytochrome P4501A (CYP1A), a drug-metabolizing enzyme found in most vertebrates, has been known to be inducible by intrinsic and extrinsic factors. CYP1A reacts specifically to environmental pollutants including polycyclic aromatic hydrocarbons (PAHs) to solubilize them to excrete from the body, and some metabolic intermediates of the environmental pollutants have been proven to be carcinogenic. PAHs induce CYP1A gene after consecutive binding to cytosolic aryl hydrocarbon receptor (AhR) and AhR nuclear translocator (ARNT) in nucleus [27].

A recent study [26] reported a significant PAH contamination in European eel caught in Moroccan rivers and lagoons by determining PAH metabolites in the bile fluid. However, no information is available on a possible PAH pollution of marine fish species in Morocco waters. Therefore, three eel species, *Anguilla anguilla*, *Conger conger* and *Muraenidae*, caught in areas with different industrialization levels were investigated.

This study aims to provide first information on concentrations of PAH metabolites of Conger eels and Moray in Morocco to compare the levels of PAH metabolites in fish from the lagoon and the open sea and to compare levels of PAH metabolites in different fish species.

## Methods

### Fish sampling

Twenty-eight Conger eels were obtained from Boujdour Sea (26°07′N, 14°29′W) in the Atlantic Ocean, 18 European eels and 7 Moray were obtained from Moulay Bousselham Lagoon in Morocco, in October 2014 and November 2015.

Moulay Bousselham Lagoon is situated on the Atlantic north coast of Morocco (34.83ºN, 6.27ºE). It is the largest protected area of the Moroccan Atlantic coast classified as “permanent biological reserve” and Ramsar site [28]. The population in Moulay Bousselham lagoon and adjacent area is about 154,000. The main activities are land cultivation and cattle farming (practiced by more than 90% of the population); artisanal fishing and shellfishing (15%) as well as summer tourism are considered as important income sources for the locals [29]. The lagoon itself covers 4500 ha, of which 30% is open water, and has an average depth of 1.5 m. The presence of heavy metals carried by the Oued Drader and Nador Canal in Merja Zerga lagoon is especially observed during floods [30]. Nador Canal and Oued Drader drain several paddy fields (culture of rice) and other crops using pesticides and agricultural inputs threatening the ecological balance of the site.

The Nador Canal carries sewage and drainage areas located on the coastal strip south of the lagoon, which represent a surface of more than 220,000 hectares.

Pesticides use and modification of the natural environment are the causes of pollution due to the diffusion of phytosanitary molecules, which degrade the quality of water resources and wetland ecosystems downstream, in particular the Merja Zerga. Studies have shown indeed the presence of high levels of pesticides in Merja Zerga [31].

Other studies regarding levels of pesticides in water and soil resources in the perimeter of Gharb conducted by ORMVAG [32] proved the worsening situation following a punctual contamination of groundwater in coastal zone by pesticide residues.

Boujdour is situated in the southern part of the Atlantic Moroccan coast, at 26° 07′ 37″N, 14° 29′ 57″W and is the name of a nearby town with a population of 41,178 inhabitants [33]. The industrial area located near the new port of Boujdour, at about 200–350 km away from Laayoune Dakhla, has offered interesting business opportunities, particularly through privileged access to fishery resources of the region.

**Fig. 1:**
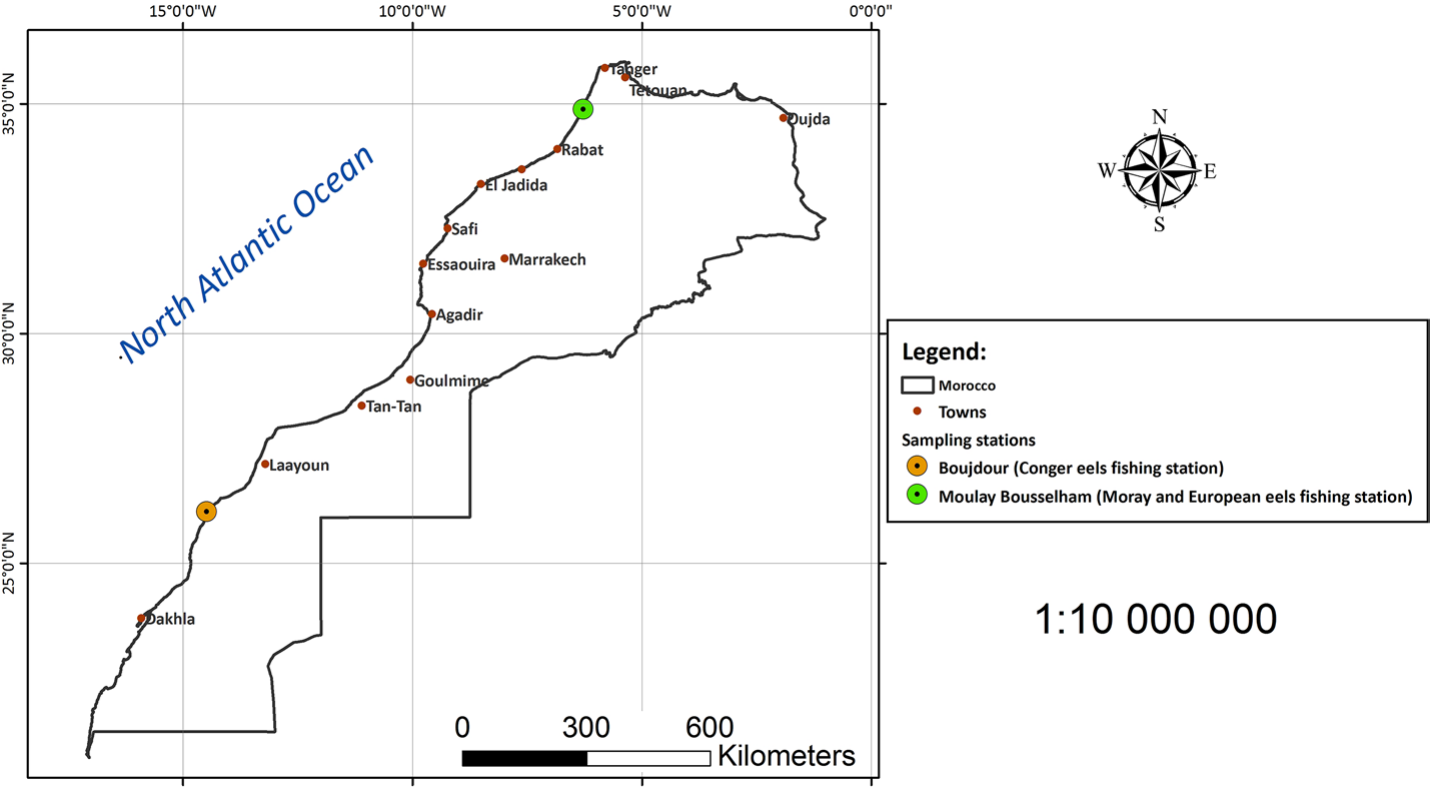
Sampling locations of Moray and European eels at Moulay Bousselham Lagoon (S1) and Conger eels at Boujdour (S2) Morocco

Sampling locations are shown in Fig. 1. Body length and weight were recorded for each fish (Table 1). After opening up the body cavity, bile fluid was collected by a disposable syringe. Bile samples were immediately frozen and stored at 18 °C or lower.

**Table 1:** Length, weight, PAH metabolites 1-hydroxypyrene (1-OH-Pyr) and 1-hydroxyphenanthrene (1-OH-Phen), bile pigments measured as absorption of bile at 380 nm of European eel, Conger eel and Moray from Moroccan waters given as mean and range (in brackets)

Species	*N* ^a^	Length (cm)	Weight (g)	1-OH-Pyr (ng/mL)	1-OH-Phen (ng/mL)	A.E. (a.u./mL)^b^
European eel	18	29.3 (22.5–46.5)	47 (20–234)	8.43 (7.34–15.56)	6.97 (5.23–9.62)	50.53 (15.31–57.19)
Conger eel	28	73.3 (56–85.5)	988 (400–1800)	0.5 (<LOD–1.63)	0.03 (<LOD–0.35)	11.77 (7.97–21.08)
Moray	7	87.3 (75.0–98.5)	1499 (750–2100)	2.97 (0.65–13.28)	0.37 (<LOD–1.14)	11.68 (5.45–23.3)

^a^Number of individuals

^b^Arbitrary units/mL

### Sample preparation and determination of PAH metabolites

PAH metabolites in bile were determined by the method of Kammann et al. [34]. In brief, 25 µL (individual or pooled sample) of fish bile was mixed with 95 µL of water and 5 µL of β-glucuronidase/arylsulfatase solution (30–60 U/mL). The mixture was subsequently incubated for 2 h at 37 °C on a heated shaker for enzymatic deconjugation. The reaction was stopped by the addition of 125 µL of ethanol. After centrifugation, the supernatants were subjected to HPLC analysis immediately. PAH metabolites 1-hydroxypyrene and 1-hydroxyphenanthrene were separated by HPLC (Lachrom System; Merck Hitachi). Samples were analyzed on a Nucleosil 100-3 C18 (3 × 125 mm) reversed phase column at a flow of 0.55 mL/min. The initial mobile phase was acetonitrile containing 0.1% trifluoroacetic acid 50/50 (v/v) changing progressively after 10 min to 60% acetonitrile over 4 min and afterwards to 100% acetonitrile within 2 min.

Detection was performed by fluorescence. Standard solutions were diluted in acetonitrile containing 5 mg/mL of ascorbic acid. The excitation/emission wavelength pairs for 1-OH-Pyr and 1-OH-Phen were 346/384 and 256/380 nm, respectively. For the quantification of bile pigments, 25 µL of bile was added to 475 µL of water and the absorbance was recorded at 380 nm in microplates (Fluostar Optima, BMG Labtech, Offenburg, Germany).

### Quality assurance and statistics

Quantification was performed using a five-point calibration. The limit of detection (LOD) was calculated using the calibration curve according to DIN 32645. The LOD for 1-OH-Pyr and 1-OH-Phen was 0.34 and 0.05 ng/mL bile, respectively. Every bile sample was analyzed twice. The recovery of 1-OH-Pyr was 98%. Certified Standard solutions of 1-OH-Pyr and 1-OH-Phen were purchased by LGC (Dr. Ehrenstorfer Standards distributed by LGC, Middlesex, UK). All other chemicals were obtained from Merck (Darmstadt, Germany).

Analysis of variance (ANOVA) was applied to test the differences between species using Tukey test (*p* ≤ 0.05) using STATISTICA 6 software.

## Results and discussion

### Differences in bile metabolites between species

The mean weight, length, concentration of 1-OH-Pyr and 1-OH-Phen as well as the absorbance at 380 nm are given for each species in Table 1. The PAH metabolites 1-OH-Pyr and 1-OH-Phen were detected in all species (Table 1). European eels had significantly higher concentrations of 1-OH-Pyr than those detected in Conger eels and Moray (*p* < 0.05; Fig. 1). The concentration of PAH metabolites ranged from <0.05 ng/mL for 1-OH-Phen to more than 15 ng/mL for 1-OH-Pyr.

**Fig. 2:**
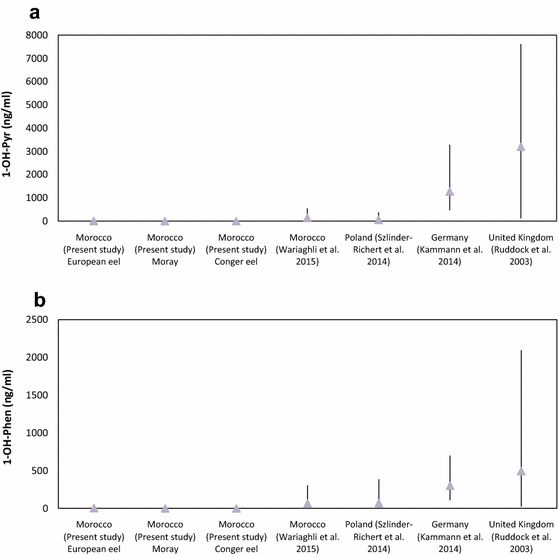
Bile metabolite 1-hydroxypyrene (**a**) and 1-hydroxyphenanthrene (**b**) concentrations detected in European eels (*Anguilla anguilla*) collected from different area and eels from Morocco (Conger, Moray and European eel) as mean (*triangles*) and range (*panels*)

The lower concentrations of 1-OH-Pyr observed in Conger eels probably reflect the less waters contamination at Boujdour coast (Table 1; Fig. 2a). Thus, the higher concentrations of PAH metabolites from European eels and Morays from Moulay Bousselham lagoon are probably due to the anthropogenic activity in this area. Comparing European eel and Moray from the same area, European eels contained significantly (*p* < 0.05) the highest metabolite concentrations; this species appears more suitable for monitoring PAH contamination. Sediments usually show higher PAH concentration levels than the water column; here pollutants are easily accumulated [35]. European eels, which spend much of their time buried in muddy sediments, will be particularly susceptible to PAH exposure [36, 37]. Accordingly, there is general agreement that sediment contamination is a major concern with regard to environmental quality. When land-disturbing activities occur, soil particles are transported by surface water movement. Soil particles transported by water are often deposited in streams, lakes, and wetlands. Sediment is the largest single nonpoint source of pollutants and the primary factor in the deterioration of surface water quality. Although the dietary preferences of European eels may result in higher exposure to PAHs than in the other species [38], the accumulation of PAHs from contaminated food is considered less efficient than uptake from the surrounding water [39].

The concentration of PAH metabolites in the fish was influenced by the different types of areas: the open sea (Boujdour) is not really polluted [40]; what appears plausible. The lagoon, as a semi-closed environment (Moulay Bousselham lagoon) presents a higher contamination than the open sea because of its lower water exchange. Here, pollutants are easily accumulated [35]. 1-OH-Pyr is invariably the major metabolite present in the bile of fish exposed to PAH contamination of sediments [41, 42], which was confirmed by our results. Pyrene is produced by many pyrolytic and petrogenic processes [43]. It is regarded the best general indicator of PAH exposure in fish [44, 45]. Although pyrene is an extremely widespread and common contaminant, the presence of other PAH metabolites in fish bile can provide additional information about the possible origin of parent compounds. In accordance with Kammann et al. [34] and Ruddock et al. [46], 1-OH-Pyr was found to be the dominant compound in eel bile. Comparing the results of the present study to similar investigations from several European countries, it is shown that the means of both PAH metabolite concentrations in eels from the different countries are higher compared to concentrations found in eels in the present study (Fig. 2). The studies cited in Fig. 2 cover the European eels caught in rivers, lakes and lagoons.

All species investigated had a lower concentration in PAH metabolites compared to others studies. Therefore, we conclude that the possible health risk of PAH contamination for Conger eels from Boujdour coast as well as Moray and European Eels from Moulay Bousselham lagoon might be low compared to different regions in Europe.

Also in comparison to the results of Wariaghli et al. [47], who investigated PAH metabolites levels in European eels from different sites in Morocco, our results are lower.

### PAH metabolites variation with sizes

The concentration of 1-OH-Pyr varies significantly with length (*p* < 0.05) for each species.

The results obtained show that the concentration of PAH metabolites does not always increase with the size, there are obviously factors which can affect the exposure of this pollutant such as species differences, age, sex, maturity and diet.

## Conclusion

Of the three species investigated, European eels contained the highest metabolite concentrations. This species appears the most suitable for monitoring PAH contamination in Moroccan water. Since the lagoon contains PAH concentrations much higher than the coastal waters. However, the overall contamination level of the eels remained low compared to other studies.

With the present study on PAH contamination in fish from Morocco, the provided information are on spatial differences of the PAH metabolites.

Quantification and identification of the metabolites in whole bile can give a rapid indication on the level of PAH contamination.
